# Occult hepatitis B virus infection in Sudan: A systematic review and meta‐analysis

**DOI:** 10.1002/jgh3.12411

**Published:** 2020-08-26

**Authors:** Khalid Eltom, Abrar Albeely, Abdel Rahim M El Hussein, Isam M Elkhidir, Khalid Enan

**Affiliations:** ^1^ Department of Microbiology and Parasitology, Faculty of Medicine University of Khartoum Khartoum Sudan; ^2^ Department of Virology, Central Laboratory Ministry of Higher Education and Scientific Research Khartoum Sudan

**Keywords:** blood donor, hemodialysis, hepatitis B, human immunodeficiency virus, meta‐analysis, occult, prevalence, renal transplant, Sudan

## Abstract

In its occult form, hepatitis B virus infection can only be detected using molecular techniques such as polymerase chain reaction, increasing the cost of the screening process. Certain population subgroups are considered to have a higher risk of transmission and reactivation of occult hepatitis B virus infection (OBI). This review aims to estimate the prevalence of OBI among these high‐risk groups in Sudan. It was conducted under the PRISMA guidelines, targeting the literature available in MEDLINE/PubMed, ScienceDirect, Google Scholar, and Cochrane Library databases. Full‐text articles published in the last 10 years that provide prevalence estimates of OBI in Sudan were examined for fulfillment of eligibility criteria. Quality assessment of selected articles was performed using the critical appraisal tool reported by Munn *et al*. Publication bias was assessed by visual examination of the funnel plot. Meta‐analysis using the random‐effects model with 95% confidence interval was used to calculate the overall and subgroup pooled prevalence of OBI. Literature search yielded a total of 717 studies, of which only 11 articles fulfilled all selection criteria. The overall pooled prevalence of OBI was found to be 15.51%, with a high level of heterogeneity. Subgroup analysis demonstrated a prevalence of 16.48% among blood donors, 13.36% among hemodialysis patients, and 12.59% among febrile patients. Evidence for possible publication bias was detected. This review provides crucial evidence for health authorities in Sudan, outlining the necessity for re‐evaluation of the current screening strategies, especially among these high‐risk groups.

## Graphical abstract

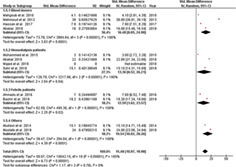

Certain population subgroups are considered to have a high risk of occult hepatitis B virus infection (OBI) transmission. This systematic review and meta‐analysis aims to estimate the prevalence of OBI among these high‐risk groups in Sudan in order to readdress the current screening strategies in the country.

## Introduction

Hepatitis B virus (HBV; a species of the genus Orthohepadnavirus and the family of Hepadnaviridae) is a hepatotropic virus that attacks the liver, causing both acute and chronic liver disease.^1^ HBV infection remains a major health problem despite the introduction of a vaccine and antiviral treatment.^2^ The World Health Organization (WHO) estimates that, in 2015, 257 million people were living with chronic HBV infection, with an estimated 887 000 deaths, resulting mostly from cirrhosis and hepatocellular carcinoma.^1^

HBV is highly infectious, even in its occult form, and remains infectious on environmental surfaces for up to 7 days.^3^ Among adults, HBV is commonly transmitted through percutaneous (i.e. puncture through the skin) or mucosal contact (i.e. direct contact with mucous membranes), as well as through exposure to infectious blood or body fluids (including semen and vaginal secretions).^3^ In addition, vertical transmission of HBV is well documented. Vertical HBV transmission usually takes place during the perinatal period; however, transplacental and postnatal transmissions were also reported.^4^, ^5^, ^6^ Furthermore, there have been some reports of vaccine failure and vertical transmission of HBV despite immunoprophylaxis, especially when the mother is highly viremic.^7^

Occult HBV infection (OBI) is defined as the presence of HBV‐DNA in the liver (with detectable or undetectable HBV‐DNA in the serum) of individuals testing HBsAg negative by currently available assays.^8^ OBI can manifest in four forms of clinical conditions: recovery from past infection indicated by the presence of hepatitis B surface antibodies (anti‐HBs); chronic hepatitis with surface gene escape mutants that are not recognized by current assays; chronic carriage without any marker of HBV infection other than HBV‐DNA (referred to as “seronegative”); and chronic carriage stage with HBsAg too low to be detected and recognized by the presence of anti‐HBc as the only serological marker (referred to as “anti‐HBc alone” or “isolated anti‐HBc”).^9^

The clinical significance of OBI includes: (i) *reactivation*: can cause fulminant hepatitis due to reactivation in immunocompromised hosts, for example, human immunodeficiency virus (HIV) patients and patients on chemotherapeutic drugs; (ii) *transmission*: potential risk of transmission of infection through blood donors, transplant donors, and hemodialysis; (iii) *hepatocellular carcinoma* (HCC): association with the development of HCC; (iv) *progression of chronic liver disease*: affects the progression of disease and response to treatment in chronic HCV patients; and (v) may be associated with cryptogenic liver disease.^10^

The prevalence of OBI is higher in regions of the world where HBV is endemic and less prevalent in regions with intermediate HBV prevalence rates. Nevertheless, certain groups are considered to have a higher risk of acquiring OBI than the general population, namely, blood transfusion recipients, liver transplant recipients, patients coinfected with hepatitis C virus/human immunodeficiency virus, patients undergoing immunosuppressive therapy or hemodialysis, patients with liver cirrhosis, cryptogenic liver disease or abnormal alanine transaminase, health‐care workers, and patients with lymphoma or rheumatoid arthritis.^10^

Many studies investigated the prevalence of OBI in certain risk groups in Sudan; however, these studies contain much variation in data and therefore require further summary and analysis for accuracy. The aim of this study is to determine the prevalence of OBI among the high‐risk groups in Sudan through a systematic literature review of published data and to make policy recommendations to control its transmission.

## Methods

### *Review protocol*

This systematic review was conducted in full compliance with the Preferred Reporting Items for Systematic Reviews and Meta‐Analyses (PRISMA) guidelines.^11^ The protocol for this systematic review was developed and registered on Open Science Framework (OSF Registries) under this digital object identifier: (10.17605/OSF.IO/TMKWJ).

In May 2020, an electronic literature search was commenced, and the following databases were used: MEDLINE/PubMed, Google Scholar, Cochrane Library, and ScienceDirect. Two reviewers formulated the search strategy. These databases were queried to search the “Titles and Abstracts” of studies for the following keywords: “occult,” “hepatitis B,” “HBV,” “OBI,” “Sudan,” and “the Sudan.” The “publication date” filter was set at 10 years. The search strategy used in PubMed is provided in supporting information. This strategy has also been adapted to identify relevant articles from other databases. All search results were managed using Mendeley reference management software, and all duplicates were removed using the same software.

### *Inclusion and exclusion criteria*

We only considered studies published between 2009 and 2019. The aim of limiting the timeline of this review to a 10‐year interval was to provide an updated, in‐depth analysis of the epidemiology OBI in Sudan, which can be replicated for comparability in the following decades. In addition, the population under study had to fit the profile of at least one of the following high‐risk groups: patients with a previous history of chronic or acute HBV infection; patients co‐infected with hepatitis C virus or human immunodeficiency virus; patients undergoing chemotherapy or anti‐CD20 therapy; recipients of organ transplant; blood donors; organ transplant donors; thalassemia and hemophilia patients; health‐care workers; patients with liver‐related disease (cryptogenic); hemodialysis patients; patients undergoing lamivudine or interferon therapy; and children at the time of the HBV vaccination. Furthermore, to minimize the risk of measurement bias, eligibility criteria also included the use of Enzyme‐Linked Immuno‐Sorbent Assay (ELISA) for HBsAg detection and conventional, nested, or real‐time polymerase chain reaction for molecular detection of HBV‐DNA. Finally, only reports written in English language were included. Exclusion criteria included all of the following: case reports, case series, editorial letters, reviews, conference abstracts, and comments.

Two reviewers independently cross‐examined the titles and abstracts of the identified studies against the inclusion and exclusion criteria. Any discrepancies were resolved by consulting a senior reviewer (Khalid Enan), and the final decision was determined only by consensus. The full texts of all eligible studies were retrieved for further assessment. Failure to retrieve the full text of a study meant its exclusion from this review.

### *Quality assessment*

Two reviewers assessed the quality and risk of bias of included studies using the critical appraisal tool for prevalence studies designed by the Joanna Briggs Institute (JBI) and reported by Munn *et al*. in 2014.^12^ This 10‐question model was designed to assess the risk of confounding bias, selection bias, and bias related to measurement and data analysis. Each question was answered either with a “yes,” “no,” “unclear,” or “not/applicable.” A study sample was considered to represent the targeted population if its basic characteristics were found to approach the true population parameters. Random (probability) sampling was considered the proper recruitment technique. The adequate sample size was calculated by reviewers using the equation *n* = *Z* ^*2*^ *p*(1 − *p*)/*e* ^2^, where *n* is the sample size, *Z* is the *Z*‐score, *p* is the prevalence estimate, and *e* is the margin of error. The adequate sample size was found to be at least 98 participants per study, using the prevalence estimate reported in central Sudan by Mudawi *et al*. in 2007 (6.8%), with 95% confidence level (*Z* = 1.96) and 5% margin of error.^13^

### *Data extraction*

Two reviewer extracted the following information from eligible articles: author(s), title, journal, year of publication, study location, population profile (i.e. the targeted risk group), demographic characteristics of the sample, number of participants, the molecular technique(s) used to detect HBV‐DNA, and the prevalence estimates of OBI.

### *Data analysis*

Each article selected for final analysis was included in a quantitative meta‐analysis to determine the overall and subgroup pooled prevalence of OBI among high‐risk groups in Sudan. Studies were weighted according to the prevalence effect size and the inverse of variance. If a study used more than one molecular technique, the prevalence estimate of the most sensitive technique was chosen to be included for further analysis. Heterogeneity analysis was carried out using the inconsistency index (I^2^) as it is known to be less influenced by the small number of articles when compared to other methods. The meta‐analysis was conducted using a generic inverse variance outcome. The prevalence estimate of each study was used as the effect estimate, while the corresponding standard error (SE) for each study was calculated using the equation *SQRT*(*p*(1 − *p*)/*n*), where *p* is the study prevalence estimate, and *n* is the number of participants in that study. The random‐effects model was used to generate summary prevalence data (displayed on forest plots) with 95% confidence interval [CI]. In addition, subgroup analysis (based on high‐risk groups) was performed to investigate for possible explanations of significant heterogeneity, and individual subgroup forest plots were generated. Finally, a funnel plot was created to assess the possibility of publication bias. All statistical analyses and figure productions were carried out using Review Manager 5.3 (The Cochrane Collaboration).^14^

## Results

### *Study selection*

**Figure 1:**
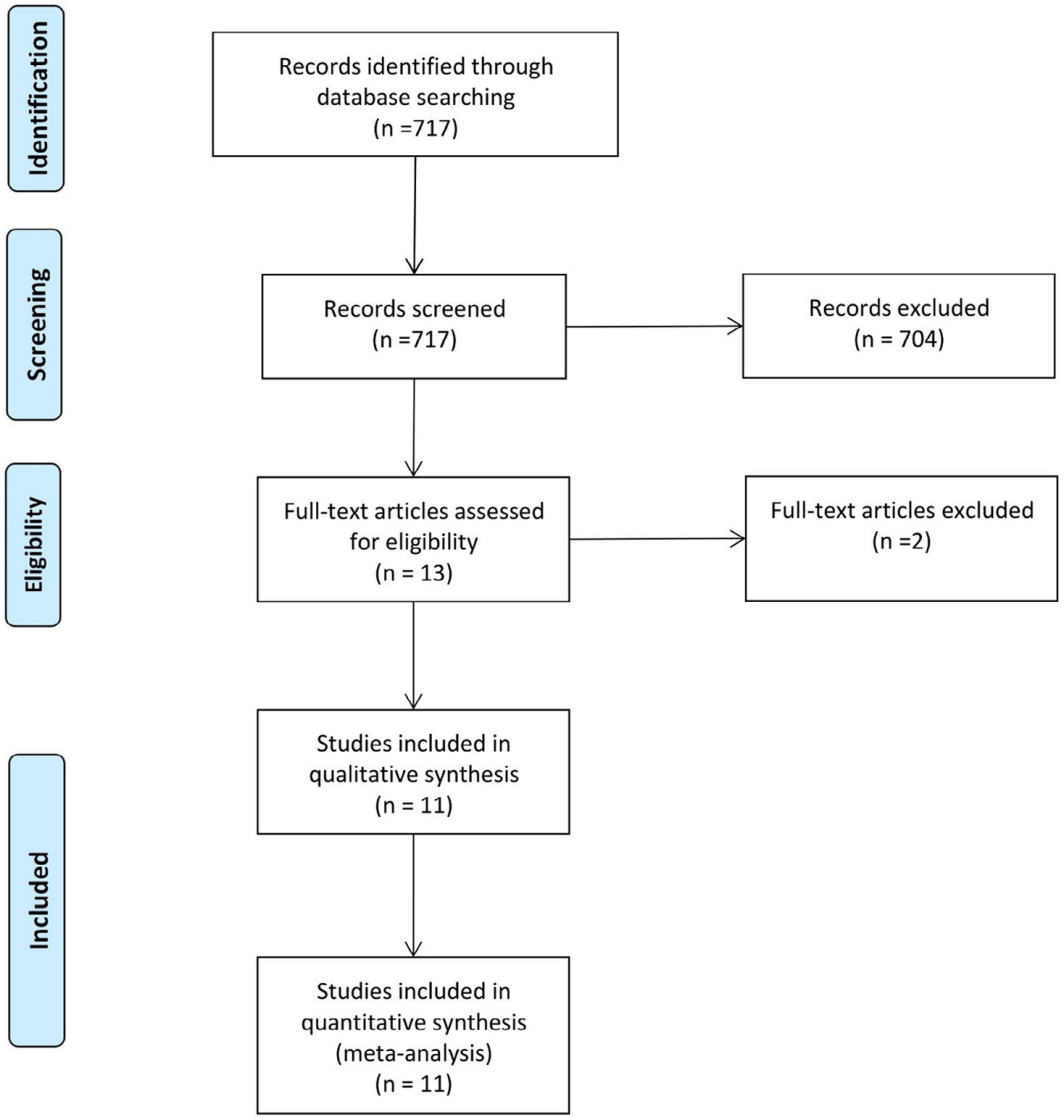
PRISMA flow chart outlining the process of article selection.

The database search yielded a total of 717 potentially relevant studies. The initial phase of removing duplicates and screening the titles and abstracts of identified studies resulted in the exclusion of 704 articles. The full texts of the remaining 13 studies were retrieved for further selection. Two studies were subsequently excluded from this review; one of them was a systematic review, and the other was excluded due to unclear methodology and sampling frame. Figure 1 outlines the process of article selection.

### *Study characteristics*

Overall, only 11 articles fulfilled all eligibility and selection criteria (Table 1). They offered a sum of 1733 participants, covering seven states in Sudan, namely, Khartoum,^15^, ^22^, ^23^, ^24^, ^25^ Gezira,^19^ River Nile,^16^ White Nile,^18^ Northern Darfur,^17^ Southern Darfur,^20^ and West Kordofan.^21^ In addition, identified studies targeted the following high‐risk groups: blood donors,^19^, ^20^, ^24^, ^25^ hemodialysis patients,^17^, ^18^, ^19^, ^22^ febrile patients,^16^, ^21^ HIV‐positive patients,^23^ HCV‐infected patients,^23^ and renal transplant recipients.^15^ Results of critical appraisal of individual studies are provided in Table 2.

**Table 1:** Data extracted from eligible studies

Author	Year of publication	State	Target group	HBV‐DNA detection technique	Sample size	Prevalence number	Prevalence estimate (%)
Mustafa *et al*.^15^	2019	Khartoum	Renal transplant recipients	PCR	100	0	0.0
				Real‐time PCR		19	19
				Nested PCR		24	24
Bashir *et al*.^16^	2019	River Nile	Febrile patients	PCR	89	1	1.1
				Real‐time PCR		16	18.2
Sahr *et al*.^17^	2019	Northern Darfur	Hemodialysis patients	PCR	90	14	15.5
Majed *et al*.^18^	2018	White Nile	Hemodialysis patients	PCR	89	0	0.0
Abakar^19^	2018	Gezira	Blood donors	Nested PCR	197	32	16
			Hemodialysis patients	Nested PCR	188	42	22
Hassan *et al*.^20^	2017	Southern Darfur	Blood donors	Nested PCR	177	14	7.9
Ahmadu *et al*.^21^	2016	West Kurdofan	Febrile patients	PCR	100	7	7.0
Mohammed *et al*.^22^	2015	Khartoum	Hemodialysis patients	PCR	100	3	3.0
Mudawi *et al*.^23^	2014	Khartoum	HIV patients	Real‐time PCR	358	54	15.1
Mahmoud *et al*.^24^	2013	Khartoum	Blood donors	Real‐time PCR	100	38	38.0
Mahgoub *et al*.^25^	2010	Khartoum	Blood donors	Real‐time PCR	145	6	4.1

HIV, human immunodeficiency virus; PCR: polymerase chain reaction.

**Table 2:** Critical appraisal (quality assessment) of selected studies

	Mustafa *et al*.^15^	Bashir *et al*.^16^	Sahr *et al*.^17^	Majed *et al*.^18^	Abakar^19^	Hassan *et al*.^20^	Ahmadu *et al*.^21^	Mohammed *et al*.^22^	Mudawi *et al*.^23^	Mahmoud *et al*.^24^	Mahgoub *et al*.^25^
Was the sample representative of the target population?†	Unclear	Unclear	Unclear	Unclear	Unclear	No	Unclear	Unclear	No	No	Unclear
Were study participants recruited in an appropriate way?^‡^	Yes	Yes	Yes	Unclear	Yes	Yes	Yes	Yes	Yes	Unclear	Unclear
Was the sample size adequate?§	Yes	No	No	No	Yes	Yes	Yes	Yes	Yes	Yes	Yes
Were the study subject and settings described in detail?	Yes	Yes	Yes	Yes	Yes	Yes	Yes	Yes	Yes	Yes	Yes
Is the data analysis conducted with sufficient coverage of the identified sample?	Yes	Yes	Yes	Yes	Yes	Yes	Yes	Yes	Yes	Yes	Yes
Were objective standard criteria used for measurement of the condition?	Yes	Yes	Yes	Yes	Yes	Yes	Yes	Yes	Yes	Yes	Yes
Was the condition measured reliably?	Yes	Yes	Yes	Yes	Yes	Yes	Yes	Yes	Yes	Yes	Yes
Was there appropriate statistical analysis?	Yes	Yes	Yes	Yes	Yes	Yes	Yes	Yes	Yes	Yes	Yes
Are all important confounding factors/subgroups/differences accounted for?	NA	NA	NA	NA	Yes	NA	NA	NA	NA	NA	NA
Were subpopulations identified using objective criteria?	NA	NA	NA	NA	Yes	NA	NA	NA	NA	NA	NA

^**†**^ Study sample was considered to represent the targeted population if its basic characteristics were found to approach the true population parameters.

^**‡**^ Random (probability) sampling was considered the proper recruitment technique.

^§^ The adequate sample size was calculated by the reviewers. It was found to be at least 98 participants per study.

NA, not applicable.

### *Overall* *OBI* *prevalence*

**Figure 2:**
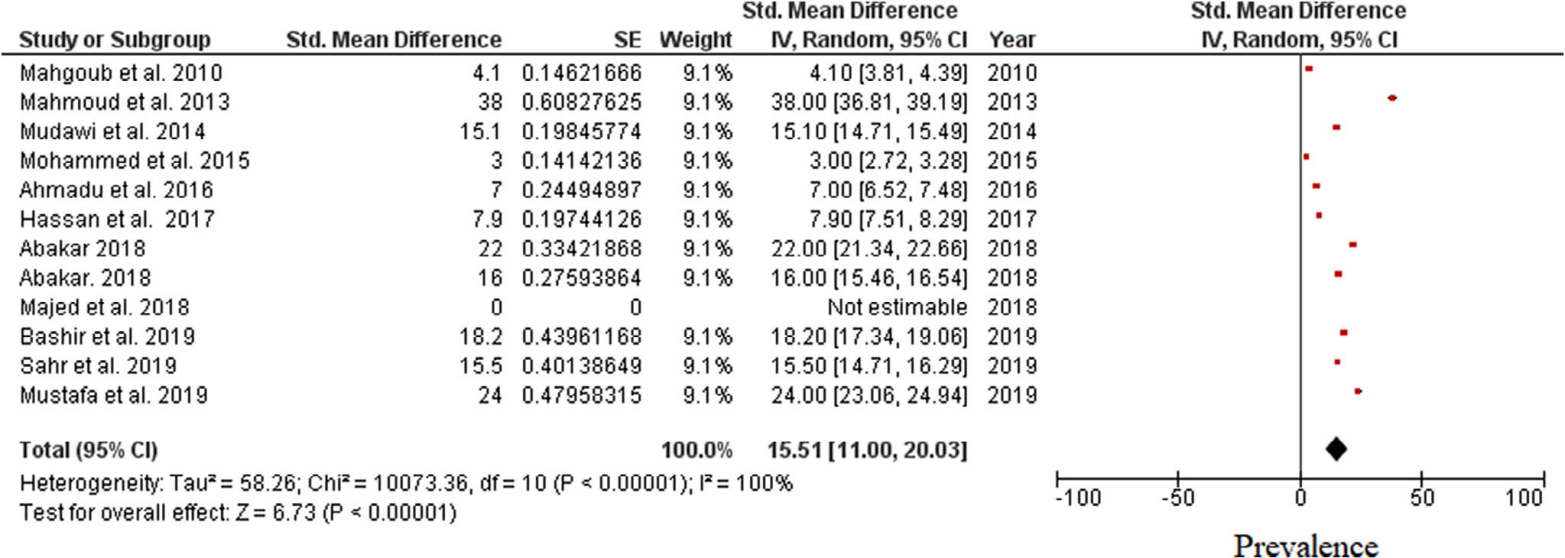
Forest plot showing overall pooled prevalence of occult hepatitis B virus infection among high‐risk individuals in Sudan.

**Figure 3:**
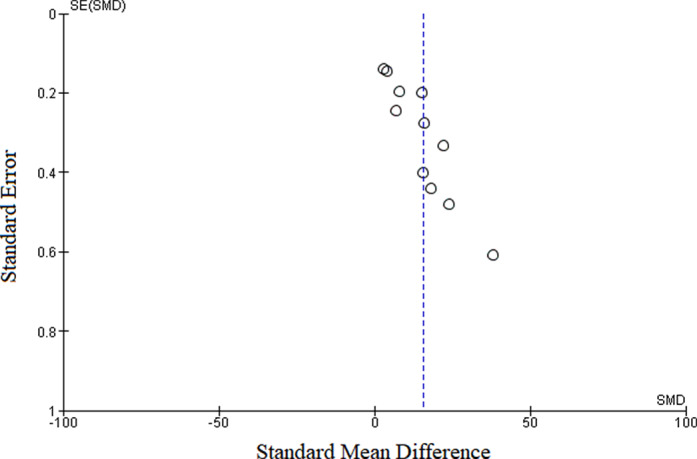
Funnel plot for assessment of publication bias. Articles were distributed asymmetrically on either side of the pooled prevalence estimate, indicating a possible publication bias.

The meta‐analysis evaluated data from all 11 articles, providing 12 prevalence estimates. The reported prevalence estimates ranged between 0 and 38% (Fig. 2). Random‐effects analysis estimated an overall pooled prevalence of OBI in Sudan of 15.51% (95% CI: 11–20%), with a significantly high level of heterogeneity (I^2^ = 100%). In visual analysis of the funnel plot, more articles were found near the top, with an asymmetrical distribution of studies on either side of the overall pooled prevalence estimate, indicating a possible publication bias (Fig. 3).

### *Subgroup analysis*

**Figure 4:**
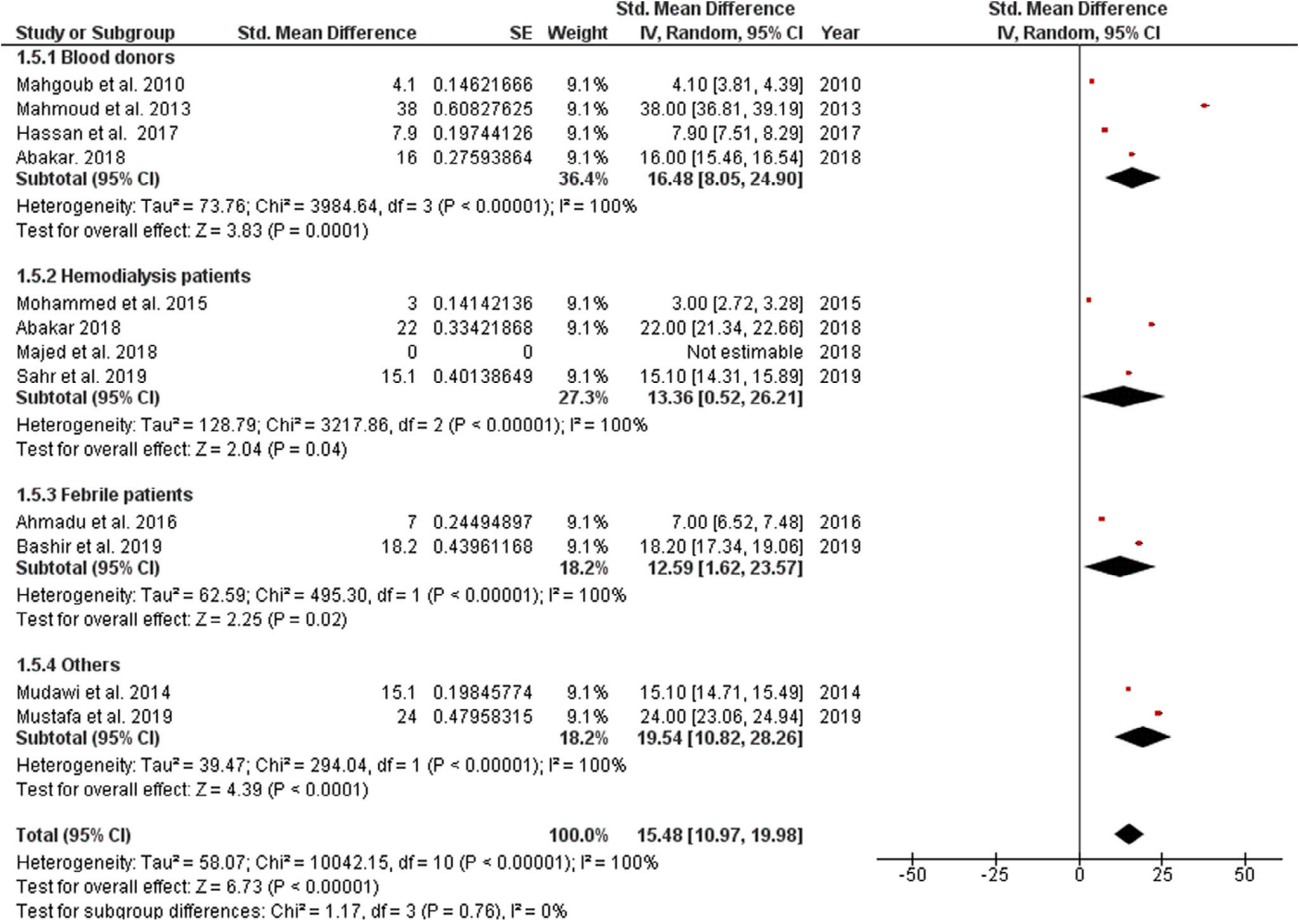
Forest plot of subgroup analysis based on specific high‐risk group for occult hepatitis B virus infection in Sudan.

All selected studies estimated the prevalence of OBI in specified high‐risk groups (Fig. 4). Meta‐analysis of studies on blood donors displayed a subgroup pooled prevalence of 16.48% (95% CI: 8.05–24.90%) and a significantly high level of heterogeneity (I^2^ = 100%). In contrast, subgroup pooled prevalence in hemodialysis patients was 13.36% (95% CI: 0.53–26.21%) with a similarly high level of heterogeneity (I^2^ = 100%). Finally, pooled prevalence of OBI among febrile patients was found to be 12.59% (95% CI: 1.62–23.57%), and the level of heterogeneity was also high (I^2^ = 100%). No subgroup analysis was performed on HIV patients and renal transplant recipients as this review could identify only a single study per each of these subgroups.

## Discussion

HBV infection is highly endemic in Sudan. This is hypothesized to be translated into a similarly significant prevalence of OBI. Studies included in this review investigated the prevalence of OBI among a number of high‐risk groups. Due to the unrealistic logistics required for the detection of OBI on a large scale (i.e. whole population), this review aimed to measure the overall and subgroup pooled prevalence of OBI among high‐risk groups, providing a relative assessment of the status of OBI in the Sudanese population.

This meta‐analysis estimates a high pooled prevalence of OBI, with 15.51% of individuals within the included high‐risk groups demonstrating a detectable level of HBV‐DNA despite being negative for HBsAg. Subgroup analysis according to high‐risk groups was conducted for studies reporting prevalence estimates in blood donors, hemodialysis patients, and febrile patients, also revealing relatively high prevalence estimates in all subgroups. The pooled prevalence of OBI among blood donors in Sudan was found to be 16.48%, which is considerably higher than the prevalence estimates reported in other countries such as Cameroon (0.56%), Japan (1.94%), and Egypt (4.1 and 4.6%).^26^, ^27^, ^28^, ^29^ On the other hand, the pooled prevalence estimate of OBI among hemodialysis patients in Sudan (13.36%) corresponds with the results in literature, which ranged from 0 to 58% in countries such as Egypt, Japan, and Brazil.^30^, ^31^, ^32^ In addition, the prevalence of OBI among HIV patients reported by Mudawi *et al*. in 2013 exceeds the prevalence estimates of OBI/HIV coinfection reported in Cameroon (5.9 and 6.9%) and Japan (6.1%).^33^, ^34^, ^35^ Similarly, renal transplant recipients in Sudan were found to demonstrate a higher prevalence of OBI than in other countries such as Korea (2.3%) and Brazil (1%).^36^, ^37^

Meta‐analysis also revealed a significant heterogeneity between the included studies. Subgroup analysis according to high‐risk groups did not provide any explanations as a similarly high level of heterogeneity was found within all subgroups (I^2^ = 100% for all subgroups) without a significant difference between them (*P* = 0.76, I^2^ = 0%). This finding indicates an even more profound basis to this heterogeneity. For instance, different molecular techniques (conventional PCR, real‐time PCR, and nested PCR) were used to detect OBI in different studies. This methodological diversity can offer an explanation for the significantly high level of heterogeneity. In addition, there are marked demographic, cultural, and ethnic variations across different population subgroups in Sudan. This also entails a similar variation in social and cultural practices, possibly affecting the transmission of HBV within these communities. Furthermore, complex dynamics of OBI transmission and pathogenesis can be found in some high‐risk groups. In HCV/HBV coinfected patients, several molecular interactions were found to suppress the replication of HBV‐DNA and the secretion of HBsAg.^38^, ^39^, ^40^, ^41^ Hemodialysis patients are considered to be at risk of acquiring both overt and occult HBV infections because of their need for multiple blood transfusions, the invasive procedures they undergo, shared dialysis equipment, impaired host immune response, and lower response rates to HBV vaccination.^32^, ^42^ Renal transplant recipients can acquire OBI via procedures such as hemodialysis, blood transfusions, and the kidney transplantation itself, which involves invasive operative procedures and an organ from a donor who might have OBI.^36^ This clinical diversity noted across different communities, geographical areas, and high‐risk groups is proposed to have a substantial influence on the heterogeneity of included studies.

The findings of this meta‐analysis have important inference for health policies, clinical practice, and research in Sudan, a country that has an estimated total of 59 000 people living with HIV, a relatively high prevalence of HCV infection (2.3% in 2015), and an annual incidence of 70–140 new cases of end‐stage renal disease (ESRD) per million inhabitants per year, with renal transplantation accounting for 28% of the total provided replacement therapies.^43^, ^44^, ^45^, ^46^ The detection of OBI is costly, especially for developing countries such as Sudan. As a result, more cost‐effective preventive strategies should be implemented. The authors propose a two‐axis approach to control the transmission of OBI in Sudan: to focus on the detection of OBI in high‐risk groups and to address the prevalence of HBV infection in the general population. The use of highly sensitive molecular techniques (e.g. real‐time PCR) for the detection of HBV‐DNA in high‐risk groups addressed by this review is extremely important, although more studies should be conducted to further support the current evidence. In addition, Sudan still demonstrates a high prevalence of HBV infection in the general population, which directly reflects a high prevalence of OBI. Consequently, more effort should be made to effectively decrease these prevalence estimates, and wider vaccination coverage would be the optimal strategy in Sudan. The authors recommend adopting the updated recommendations of the Advisory Committee on Immunization Practices (ACIP) and Centers for Disease Control and Prevention (CDC) on prevention of HBV infection in 2018.^3^

This systematic review and meta‐analysis has its limitations. First, we identified prevalence estimates in five high‐risk groups only. This can hinder the generalization of our findings. The significant heterogeneity and evidence of publication bias detected in this meta‐analysis can also affect the reliability of these results. Furthermore, all studies were found to merely report prevalence estimates of OBI, with very poor emphasis on the demographic and clinical characteristics of the study samples and the targeted populations. Accordingly, we were unable to fully explore and analyze the clinical spectrum and sociodemographic characteristics of OBI in Sudan.

## Conclusion

OBI is a serious public health concern. Its clinical significance stems from the possibility of its transmission and reactivation, in addition to its potential contribution to the development of progressive liver disease and HCC. Sudan was found to have a high prevalence of OBI among high‐risk groups, specifically blood donors, hemodialysis patients, renal transplant recipients, HIV patients, and febrile patients. More research is needed to strengthen the current evidence and to describe the prevalence of OBI among other high‐risk groups. In addition, health authorities in Sudan should seriously address the high prevalence of OBI in this country. Preventive strategies should be meticulously designed and implemented in order to control its transmission and possible complications.

## Supporting information


**Appendix** S1. Supporting information.
