# Supplementary material for: Occult hepatitis B virus infection in Sudan: A systematic review and meta‐analysis
Source: JGH Open. 2020 Aug 26;4(5):800–7. doi: 10.1002/jgh3.12411 (PMC7578306; doi:10.1002/jgh3.12411)
Supplement: Supplementary file 1 — Appendix S1. Supporting information. [file JGH3-4-800-s001.pdf]

Search strategy in PubMed

Query box:

(("occult"[Title/Abstract]) OR ("OBI"[Title/Abstract])) AND (("hepatitis B"[Title/Abstract]) OR ("HBV"[Title/Abstract])) AND (("Sudan"[Title/Abstract]) OR ("the Sudan"[Title/Abstract]))

Filters:

Publication date= 10 years.
